# Analysis of meiotic segregation modes in biopsied blastocysts from preimplantation genetic testing cycles of reciprocal translocations

**DOI:** 10.1186/s13039-019-0423-7

**Published:** 2019-02-26

**Authors:** Jie Wang, Dong Li, Zhipeng Xu, Zhenyu Diao, Jianjun Zhou, Fei Lin, Ningyuan Zhang

**Affiliations:** Reproductive Medical Center, Drum Tower Hospital Affiliated to Nanjing University Medical College, Zhongshan Road 321, Nanjing, 210008 China

**Keywords:** Reciprocal translocation, Preimplantation genetic testing for chromosomal structural rearrangements (PGR-SR), Blastocyst trophectoderm biopsy, Next generation sequencing (NGS), Meiotic segregation mode

## Abstract

**Purpose:**

To analyse the meiotic segregation modes of chromosomal structural rearrangements (PGT-SR) of reciprocal translocation in biopsied blastocysts from preimplantation genetic testing and to investigate whether any features of reciprocal translocation, such as carrier gender or the presence of acrocentric chromosomes or terminal breakpoints, affect meiotic segregation modes.

**Methods:**

Comprehensive chromosomal screening was performed by next generation sequencing (NGS) on 378 biopsied blastocysts from 102 PGD cycles of 89 reciprocal translocation carriers. The segregation modes of a quadrivalent in 378 blastocysts were analysed according to the carrier’s gender, chromosome type and the location of chromosome breakpoints.

**Results:**

The results showed that 122 out of 378 blastocysts (32.3%) were normal or balanced, 209 (55.3%) were translocated chromosomal abnormalities, and 47 (12.4%) were abnormalities of non-translocated chromosomes. The proportion of translocated chromosomal abnormalities in translocations without acrocentric chromosomes was significantly higher than that in blastocysts from carriers with acrocentric chromosomes (14.8% versus 5.9%, *P* = 0.032). Translocation with acrocentric chromosomes exhibited a significantly higher proportion of 3:1 segregation (24.8% versus 5.1%, *P* < 0.0001) and a lower rate of 2:2 segregation (70.3% versus 87.0%, *P* = 0.00028) compared with the proportions in blastocysts from carriers without acrocentric chromosomes. The frequency of adjacent-2 segregation was significantly different in translocations with terminal breakpoints compared to the frequency in blastocysts from carriers without terminal breakpoints (6.7% versus 15.5%, *P* = 0.013).

**Conclusions:**

This study indicates that the segregation modes in blastocysts were affected by the presence of acrocentric chromosomes and terminal breakpoints, but not by the carrier’s sex. Our data may be useful for predicting the segregation pattern of a reciprocal translocation and could support genetic counselling for balanced translocation carriers for PGT cycles using blastocyst biopsy.

## Background

Reciprocal translocations are the most common structural chromosomal abnormalities, which result from the exchange of terminal segments from different chromosomes. Such translocations occur in 0.14% of the neonatal population and are found in 0.6% of infertile couples [1, 2]. Balanced reciprocal translocation carriers possess no numerical genetic material abnormalities and most are phenotypically normal. However, they have a high risk of recurrent spontaneous abortions or birth of affected children, which is due to chromosomally abnormal embryos as a result of the production of unbalanced gametes by the carriers. During meiosis I, the translocated chromosomes and their normal homologues form a quadrivalent structure that segregates via five theoretical modes: alternate, adjacent-1, adjacent-2, 3:1 or 4:0. With the occurrence of recombination, 32 kinds of gametes can be generated [3]. Only two normal/balanced gametes are produced from the alternate segregation mode, and the others are chromosomally unbalanced.

There is an extensive diversity of unbalanced gamete frequencies, ranging from 19.0 to 91.0%, among reciprocal translocation carriers [4–7]. The variability in the frequencies of the different segregation modes depends on the specific characteristic of the translocations, including the presence of acrocentric chromosomes, the position of the breakpoints and the sex of the carrier. It has been reported that the incidence of alternative segregation with acrocentric chromosomes was significantly lower than that in carriers without acrocentric chromosomes [5]. The carrier’s gender has also been found to affect meiotic segregation [6, 8]. Several studies indicated that the location of the breakpoints could also affect segregation [7, 9, 10].

However, these previous studies were performed using fluorescence in situ hybridization (FISH). The FISH method is only useful for detecting a limited number of chromosomes, and it is difficult to precisely locate the translocated chromosomal breakpoints. Next generation sequencing (NGS) is being increasingly applied in preimplantation genetic testing (PGT). NGS is a comprehensive, precise and cost-effective genetic technique that has been used to screen all 24 human chromosomes [11]. Studies conducted on trophectodermal biopsies have demonstrated that NGS can accurately detect aneuploidy and unbalanced rearrangements [12, 13]. Moreover, most of the previous information concerning meiotic segregation in preimplantation embryos formed by the gametes of reciprocal translocation carriers comes from analyses of the developmental cleavage stage (3 days post-fertilisation) [4–11, 14]. The cytogenetic segregation mode in blastocysts from reciprocal translocation carriers has only been superficially investigated.

In this study, we evaluated the meiotic segregation modes in blastocysts from reciprocal translocation carriers via comprehensive analysis with the use of NGS.

## Methods

### Study patients

This study included a total of 89 couples with balanced reciprocal translocations who had 102 PGT cycles at Nanjing Drum Tower Hospital from January 2016 through December 2017. The patients carrying reciprocal translocations were identified based on G-banded metaphase spreads obtained from peripheral blood using standard techniques. Written informed consent was obtained from each family before the start of the PGT cycles.

### In vitro fertilisation, embryo culture, trophectoderm (TE) biopsy and comprehensive chromosomal screening

Controlled ovarian hyperstimulation (COH) was performed using gonadotrophin-releasing hormone (GnRH) agonist or antagonist, recombinant follicle stimulating hormone (FSH) and human chorionic gonadotrophin (hCG). Oocytes were retrieved transvaginally under ultrasound guidance 35 h after hCG administration. Intracytoplasmic sperm injection (ICSI) was performed on retrieved MII oocytes. The two pronuclei were observed in the injected oocytes at 16-18 h post-insemination. The embryos were cultured in sequential media (G1 and G2, Vitrolife, Goteborg, Sweden) at 37 °C in a humidified atmosphere with 6% CO_2_, 5% O_2_ and 89% N_2_.

On the third morning post-insemination, a laser was used to cut an 11-um hole in the zona pellucida of the embryos that were selected for blastocyst culture. The blastocyst score was determined using Gardner blastocyst grading scale [15, 16]. Grade 5 and grade 6 blastocysts with morphology scores better than 5CC or 6CC were used for biopsy. 5CC and 6CC blastocysts were excluded for biopsy. 5AA, 5AB, 5BA, 5 AC, 5CA, 5BB, 5 BC, 5CB (the same for grade 6) were used for biopsy. Approximately 5–10 trophectoderm (TE) cells were aspirated into a biopsy pipette with a 20-um internal diameter and dissected with a laser. The biopsied TE cells were transferred into 200-uL PCR tubes for whole genome amplification (WGA).

In 2016, the biopsied TE cells were subjected to WGA using the REPLI-g Single Cell Kit (Qiagen, Valencia, CA), and in 2017, the WGA of the biopsied samples was performed using the SurePlex WGA Kit (Illumina, San Diego, USA); in both cases, the protocols were performed according to the manufacturers’ instructions. The NGS and comprehensive chromosomal screening were performed as previously described [11, 17]. The biopsied blastocysts were cryopreserved in liquid nitrogen.

### Frozen normal/balanced blastocyst transfer

The blastocyst transfer cycles included a total of 44 couples with balanced reciprocal translocations who had PGT cycles from January 2016 to December 2017. The transfer cycles were also performed from January 2016 to December 2017. The vitrified-warmed blastocyst was transferred into the uterine cavity on day 6 of progesterone administration. 14 days after blastocyst transfer, the serum hCG level was measured. Clinical pregnancy was defined by the detection of a gestational sac and a foetal heartbeat via sonography at 6 weeks after transfer.

### Statistical analysis

All statistical calculations were performed using SPSS software (version 22.0; IBM Corp, Armonk, NY), and the quantitative data plotting was performed using Prism software (version 5; GraphPad Software Inc., La Jolla, CA). The χ^2^ test was used to compare the differences between the frequency distributions of the segregation modes. Quantitative clinical characteristics were compared with Student’s t-test or the Mann-Whitney U test. *P* < 0.05 was considered statistically significant.

## Results

### Clinical outcomes of reciprocal translocation carriers

In this study, we analysed the clinical outcome of 102 preimplantation genetic testing (PGT) cycles in 89 reciprocal translocation carriers from January 2016 through December 2017. The results are presented in Table 1. One thousand five hundred fourteen cumulus-oocyte-complexes were retrieved, and ICSI was performed on 1175 mature metaphase-II oocytes. Nine hundred ninety-eight oocytes (84.9% of the total injected oocytes) were fertilized (indicated by the presence of 2-pronuclei). Nine hundred thirty-one embryos were used for blastocyst culture. Biopsies were performed on 378 blastocysts (40.6% of the total cultured embryos). Genome-wide copy number variants were successfully analysed in all of the biopsied blastocysts via next-generation sequencing. One hundred twenty-two out of 378 blastocysts (32.3%) were normal or balanced, 209 (55.3%) were translocation chromosomal abnormalities, and 47 (12.4%) were abnormalities in non-translocation chromosomes. From January 2016 to December 2017, 44 blastocysts identified as normal/balanced from reciprocal translocation carriers were transferred into the uterine cavity. Positive hCG results were obtained in 32 cycles (72.7%), and 29 deliveries (65.9%) were achieved. There were two spontaneous abortions. The karyotypes of the two abortus were normal or balanced.

**Table 1:** Clinical outcomes of PGT for reciprocal translocation

Parameter	Population
Patients	89
Cycles	102
Female age (years)	28.8±3.5
Male age (years)	30.2±4.7
Retrieved oocytes	1514
Injected oocytes	1175 (77.6%)
2-Pronuclei zygotes	998 (84.9%)
Embryos used for blastocyst culture	931
Biopsied blastocysts	378 (40.6%)
Diagnosed blastocysts	378 (100%)
Normal/balanced blastocysts	122 (32.3%)
Translocation chromosomes abnormal embryos	209 (55.3%)
Non-translocation chromosomes abnormal embryos	47 (12.4%)
Blastocyst transfer cycles	44
Positive hCG	32 (72.7%)
Biochemical pregnancies	1 (2.3%)
Clinical pregnancies	31 (70.5%)
Spontaneous abortions	2 (4.5%)
Deliveries	29 (65.9%)

Values are n, n (%) or mean±SD

*PGT* preimplantation genetic testing

The meiotic segregation mode was analysed in 378 blastocysts. Overall, 2:2 segregation was observed in 312 blastocysts (82.5%), 3:1 segregation in 39 blastocysts (10.3%) and 4:0 segregation in only one blastocyst (0.3%). Chaotic segregation modes that could not be characterized were found in 26 blastocysts (6.9%).

Some features of the reciprocal translocations, such as the presence of acrocentric chromosomes, the positions of the breakpoints and carrier gender were further investigated. The incidence of 4:0 segregation was extremely low; therefore, the study focused on the 2:2 and 3:1 segregation modes.

### Comparison of the meiotic segregation modes of the reciprocal translocations in female and male carriers

This study evaluated the embryonic development and meiotic segregation modes in biopsied blastocysts according to the gender of the translocation carriers. Sixty-one cycles in 51 couples involved male carriers and 41 cycles in 38 couples involved female carriers. The characteristics of the patients are described in Table 2.

**Table 2:** Clinical characteristics and results of embryo in vitro culture for female and male reciprocal translocation carriers

Parameter	Female carriers	Male carriers	*P* value
Patients	38	51	
Cycles	41	61	
Female age (years)	28.9±3.1	28.8±3.9	0.811^a^
Female BMI (kg/m2)	22.5±2.7	22.8±3.4	0.625 ^a^
Male age (years)	30.4±5.0	30.0±4.6	0.717 ^a^
Retrieved oocytes	14.6±7.4	15.0±6.9	0.758 ^b^
Injected oocytes	11.3±5.1	11.7±5.0	0.829 ^b^
2-Pronuclei zygotes	9.8±5.1	9.8±4.5	0.883 ^b^
Normal fertilisation rate (%)	86.9 (403/464)	83.7 (595/711)	0.161 ^b^
Embryos used for blastocyst culture	9.2±5.1	9.1±4.4	0.937 ^b^
Biopsied blastocysts	4.2±2.5	3.4±2.3	0.084 ^b^
High-quality blastocyst formation rate (%)	45.6 (172/377)	37.2 (206/554)	**0.012** ^**c**^

Values are n, n (%) or mean±SD

^a^t-text, ^b^ Mann-Whitney U test, ^c^ χ^2^ test. The bold values meant there existed significant difference

**Fig. 1:**
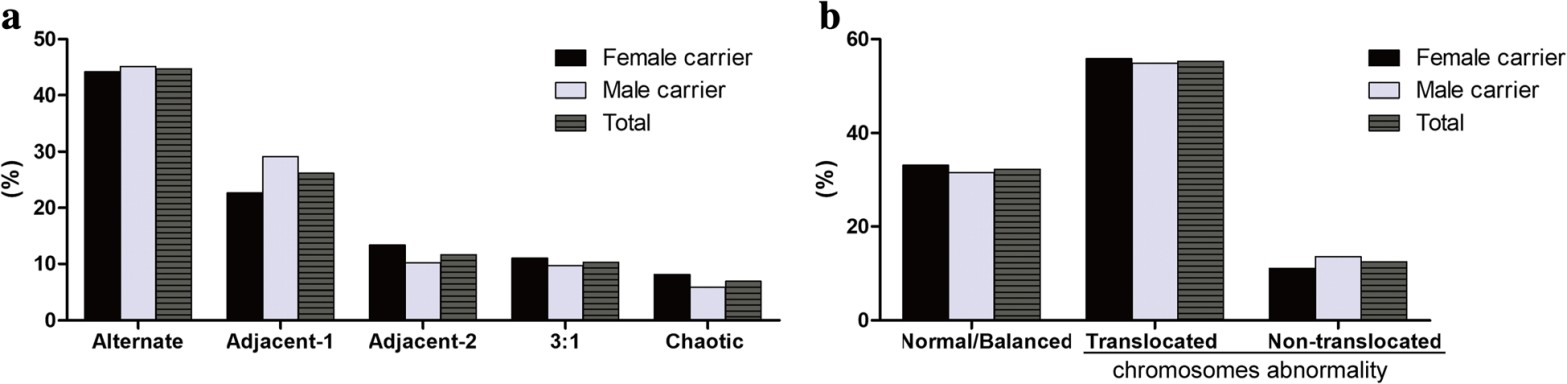
Meiotic outcomes of biopsied blastocysts from female and male reciprocal translocation carriers (**a**) Segregation modes of biopsied blastocysts; (**b**) Frequencies of normal/balanced and abnormal blastocysts

The meiotic segregation modes in the biopsied blastocysts were analysed. Although no statistically significant difference was observed between the female and male carriers for the incidence of alternate segregation (44.19% versus 45.15%), adjacent-2 segregation (13.37% versus 10.19%) and 3:1 segregation (11.05% versus 9.71%), the incidence of adjacent-1 segregation (22.67% versus 29.13%) was lower in female carriers than in male carriers (Fig. 1a). Overall, the frequencies of normal/balanced, translocated chromosome and non-translocated chromosome abnormal embryos were similar between the two groups (Fig. 1b).

### Comparison of the meiotic segregation modes of reciprocal translocations with or without acrocentric chromosomes

Twenty-five cycles in 24 carriers with acrocentric chromosomes and 77 cycles of 65 carriers without acrocentric chromosome were analysed. The mean ages of the female and male partners were similar between the two groups. The fertilization and high-quality blastocyst formation rates were identical between the two groups (Table 3).

**Table 3:** Clinical characteristics and results of embryo in vitro culture for reciprocal translocation carriers with or without acrocentric chromosomes

Parameter	With acrocentric chromosome	Without acrocentric chromosome	*P* value
Patients	24	65	
Cycles	25	77	
Female age (years)	28.5 ± 3.9	28.9 ± 3.5	0.625 ^a^
Female BMI (kg/m2)	22.4 ± 3.5	22.8 ± 3.0	0.544 ^a^
Male age (years)	29.8 ± 5.5	30.3 ± 4.5	0.632 ^a^
Retrieved oocytes	15.4 ± 7.1	14.6 ± 7.1	0.604 ^b^
Injected oocytes	12.7 ± 6.0	11.1 ± 4.6	0.349 ^b^
2-Pronuclei zygotes	10.9 ± 5.4	9.4 ± 4.5	0.290 ^b^
Normal fertilisation rate (%)	85.8 (272/317)	84.6 (726/858)	0.679 ^b^
Embryos used for blastocyst culture	10.1 ± 5.3	8.8 ± 4.4	0.333 ^b^
Biopsied blastocysts	4.0 ± 2.7	3.6 ± 2.3	0.601 ^b^
High-quality blastocyst formation rate (%)	39.9 (101/253)	40.9 (277/678)	0.855 ^c^

Values are n, n (%) or mean±SD

^a^t-text, ^b^ Mann-Whitney U test, ^c^ χ^2^ test

**Fig. 2:**
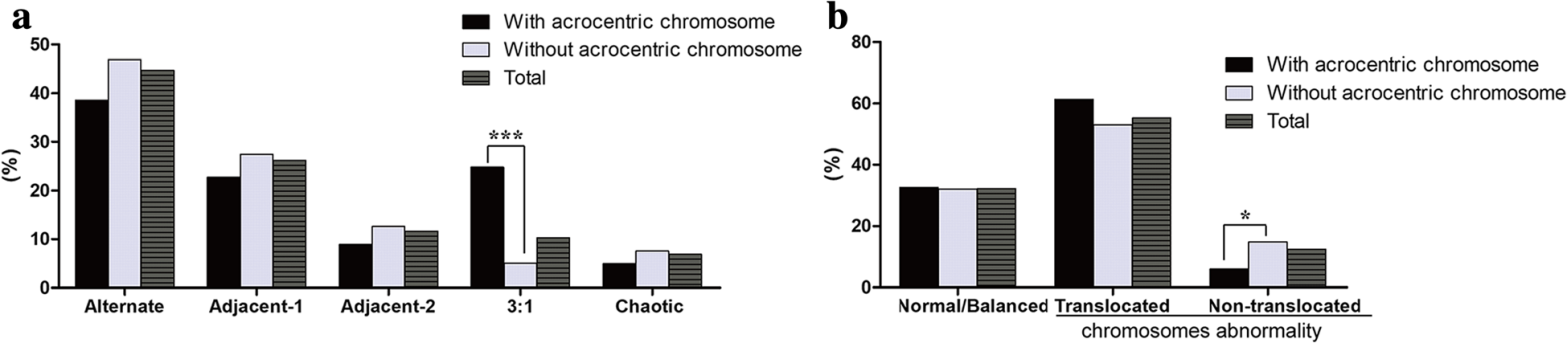
Meiotic outcomes of biopsied blastocysts from reciprocal translocation carriers with or without acrocentric chromosomes (**a**) Segregation modes of biopsied blastocysts; (**b**) Frequencies of normal/balanced and abnormal blastocysts. *P < 0.05, *** *P* < 0.001

Although the frequency of normal/balanced karyotypes in translocations with acrocentric chromosomes was not statistically significantly different from that without acrocentric chromosomes, the proportion of non-translocated chromosomal abnormalities in translocations without acrocentric chromosomes was significantly higher than that with acrocentric chromosomes (14.8% versus 5.9%, *P* = 0.032) (Fig. 2b). The translocations with acrocentric chromosomes exhibited a significantly higher frequency of 3:1 segregation (24.8% versus 5.1%, *P* < 0.0001) and a lower frequency of 2:2 segregation (70.30% versus 87.0%, *P* = 0.00028) than in cases without acrocentric chromosomes (Fig. 2a).

### Comparison of the meiotic segregation modes of reciprocal translocations with or without terminal breakpoints

A translocation with terminal breakpoints was defined as a translocation when at least one of the two translocated segments (TS)/arms involved in the translocation (ARM) had a length ratio of < 0.2. Forty-six cycles in 40 carriers with terminal breakpoints and 56 cycles in 49 carriers without terminal breakpoints were analysed. The mean age of the female patients was higher in the group with reciprocal translocations with terminal breakpoints than in the female patients in the group without terminal breakpoints. Furthermore, the female BMI showed a difference between the two groups. The male mean age was similar between the two groups. The fertilization and high-quality blastocyst formation rates were also identical in the two groups (Table 4).

**Table 4:** Clinical characteristics and results of in vitro cultured embryos for reciprocal translocation carriers with or without terminal breakpoints

Parameter	With terminal breakpoint	Without terminal breakpoint	*P* value
Patients	40	49	
Cycles	45	57	
Female age (years)	29.9 ± 3.7	27.9 ± 3.2	**0.004** ^a^
Female BMI (kg/m2)	22.0 ± 3.2	23.2 ± 2.9	**0.043** ^a^
Male age (years)	30.8 ± 4.9	29.6 ± 4.6	0.224 ^a^
Retrieved oocytes	13.6 ± 5.8	15.8 ± 7.8	0.216 ^b^
Injected oocytes	10.4 ± 4.5	12.4 ± 5.3	0.083 ^b^
2-Pronuclei zygotes	8.9 ± 4.4	10.5 ± 4.9	0.129 ^b^
Normal fertilisation rate (%)	85.7(401/468)	84.4(597/707)	0.617 ^b^
Embryos used for blastocyst culture	8.1 ± 4.2	9.9 ± 4.9	0.072 ^b^
Biopsied blastocysts	3.5 ± 2.2	3.8 ± 2.6	0.721 ^b^
High-quality blastocyst formation rate (%)	39.9(159/366)	38.8(219/565)	0.176 ^c^

Values are n, n (%) or mean±SD

^a^t-text, ^b^ Mann-Whitney U test, ^c^ χ^2^ test. The bold values meant there existed significant difference

**Fig. 3:**
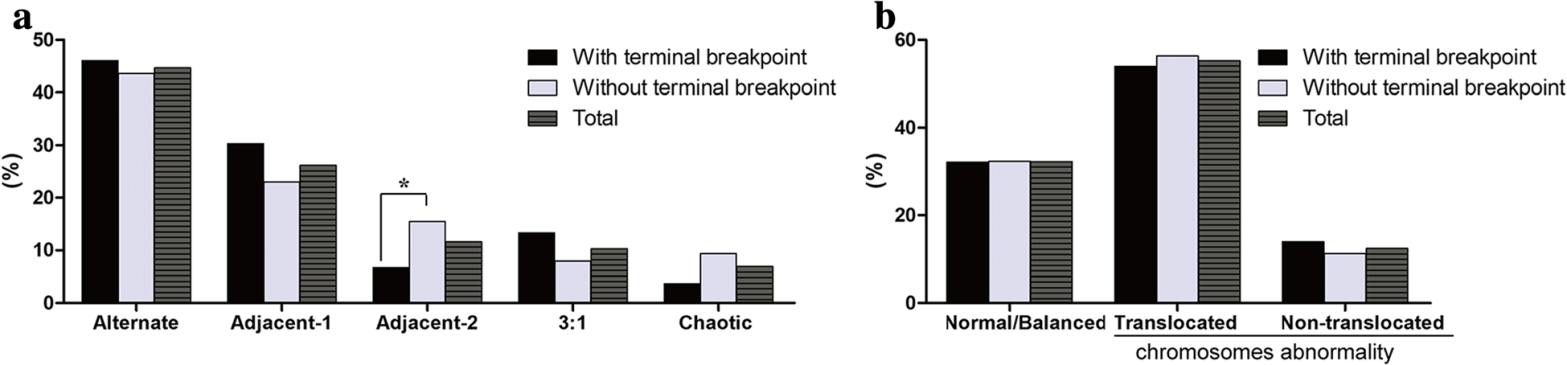
Meiotic outcomes of biopsied blastocysts from reciprocal translocation carriers with or without terminal breakpoints (**a**) Segregation modes of biopsied blastocysts; (**b**)Frequencies of normal/balanced and abnormal blastocysts. ** *P* < 0.01

The incidences of alternate (46.06% versus 43.66%), adjacent-1(30.3% versus 23.0%) and 3:1(13.33% versus 7.98%) segregation were not significantly different in cases with translocations with terminal breakpoints compared with those in cases without terminal breakpoints. The frequency of adjacent-2 segregation in cases of translocation with terminal breakpoints was significantly lower than that in those without terminal breakpoints (6.67% versus 15.5%, *P* = 0.0127) (Fig. 3a). The frequencies of embryos with normal/balanced, translocated chromosomes and non-translocated chromosomes abnormal were not different between the two groups (Fig. 3b).

## Discussion

In the present study, the chromosomal CNVs of 378 biopsied blastocysts from reciprocal translocation carriers were evaluated and the meiotic segregation modes were analysed. Our results showed a higher prevalence of alternate segregation products, followed by adjacent-1, adjacent-2, 3:1 and other chaotic segregation products. The percentage of normal/balanced embryos in biopsied blastocysts from our study was much higher than that in day 3 cleavage stage embryos from other two studies (32.3% versus 18.7% [6] and 12.6% [7]). It’s reported that chromosomal abnormalities were common even in embryos with the best morphological cleavage stage [18]. Aneuploid cleavage embryos exhibited lower chance to develop normally to the blastocyst stage [19]. The rate of identifying transferable embryos was much higher in the biopsied blastocyst PGT cycles in our study compared with the biopsied blastomeres cycles from other studies. Only two normal/balanced gamete types are produced from the alternate segregation mode; however, alternate segregation can coexist with aneuploidy on non-translocated chromosomes. After comprehensive chromosomal screening of blastocysts by NGS, reciprocal translocation carriers who were transplanted with tested blastocysts obtained higher clinical pregnancy (70.5%) and live birth rates (65.9%) in our centre (Table 1).

To clarify the features of reciprocal translocation that have effects on the meiotic segregation pattern in blastocysts, the segregation modes were analysed based on the carrier’s gender, and the presence of acrocentric chromosomes and terminal breakpoints. The incidence of alternate segregation was similar between male and female translocation carriers, which was consistent with the results of other studies [6–8] [20]. The incidences of each segregation pattern were also similar in our study, while the frequencies of the adjacent-1, adjacent-2, or 3:1segregation modes were significantly different in other studies. However, their results were contradictory. For example, the Ogilvie group reported that adjacent-2 segregation was more common in male carriers than in female carriers [21], and two other groups both reported that adjacent-2 segregation was significantly less common in male carriers compared with females [6, 7]. In a recent publication on a large cohort (1842 blastocysts) analysed by SNP array, the results showed that the rate of adjacent-1 segregation was significantly higher in male carriers than in female carriers, while the rates of the adjacent-2 and 3:1 segregation modes were lower in the male carriers [20]. Our results also showed this trend, although it was not statistically significant. During meiotic segregation, translocations including an acrocentric chromosome form an unstable quadrivalent. Lim’s group reported that the rate of alternate segregation was statistically low in reciprocal translocations with acrocentric chromosomes [5]. The present study showed a similar trend, as the proportion of alternate segregation in the biopsied blastocysts with reciprocal translocations involving an acrocentric chromosome was lower than that in blastocysts from carriers without an acrocentric chromosome; however, this trend was not statistically significant. Translocations with acrocentric chromosomes exhibited a significantly higher incidence of 3:1 segregation, which was consistence with studies from the Lim [5] and Ye [7] groups. It was known that gametes and embryos with translocations with terminal breakpoints have high frequencies of chromosomal abnormalities [7, 22], and a tendency towards increased frequencies of adjacent-2 and 3:1 segregation [7]. However, our results showed that the rate of alternate segregation in translocations with terminal breakpoints was not different from that in translocations without terminal breakpoints. The proportion of adjacent-2 segregation was lower in translocations with terminal breakpoints than that in translocations without terminal breakpoints. In our study, NGS technology allowed screening of genome-wide variants. All aspects of the chaotic segregation products and non-translocated chromosomal abnormalities could be detected. These results were consistent with Zhang’s study, which showed a higher frequency of chaotic segregation patterns and abnormal non-translocated chromosomes [20]. The differences in the results in previous studies and the present study might be caused by differences in sample number, biopsied stage or the specific methods used in each study.

The phenomenon of interchromosomal effect (ICE) might disturb proper pairing and disjunction of other chromosomes during meiosis I, which could lead to non-translocated chromosomal numerical abnormalities [23]. Our study showed that the proportion of non-translocated chromosomal abnormalities was significantly higher in translocations without acrocentric chromosomes than in translocations with acrocentric chromosomes, suggesting that ICE might be affected by the chromosome types involved in the translocation. Further study of the ICE on reciprocal translocation is required.

## Conclusion

This study suggests that the segregation modes in blastocysts were affected by the involvement of acrocentric chromosomes and terminal breakpoints, but not by the carrier’s sex. Our data may be useful for predicting the segregation pattern of reciprocal translocations and may support the use of blastocyst biopsy in the genetic counselling of balanced translocation carriers for PGD-SR cycles.
